# Is There a Need for Recovery Room Radiographs Following Uncomplicated Primary Total Knee Arthroplasty?

**DOI:** 10.7759/cureus.14544

**Published:** 2021-04-18

**Authors:** Thomas A Novack, Jay N Patel, Justin Koss, Christopher Mazzei, Colin J Harrington, James C Wittig, John Dundon

**Affiliations:** 1 Orthopedics, St. Joseph's Regional Medical Center, Paterson, USA; 2 Orthopedics, Morristown Medical Center, Morristown, USA; 3 Orthopedics, Walter Reed National Military Medical Center, Bethesda, USA; 4 Orthopedic Surgery, Orthopedic Institute of New Jersey, Morristown, USA

**Keywords:** knee arthroplasty, radiology

## Abstract

Introduction

Total knee arthroplasty (TKA) is one of the most common orthopedic procedures performed in the United States. Obtaining radiographs in the post-anesthesia care unit (PACU) has been the standard of care at most hospitals. The purpose of this study was to examine the utility and cost-effectiveness of immediate, postoperative radiographs in regards to operative decision-making to prevent complications within 90 days after primary TKA.

Methods

A retrospective review of 4,830 consecutive patients who underwent cemented or uncemented TKA between January 2016 and June 2019 at a large, regional medical center was performed. International Classification of Diseases, Tenth Revision (ICD-10) codes were used to track any readmissions within 90 days of TKA. If readmission was for a mechanical complication, including fracture, dislocation, or component loosening, PACU radiographs were reviewed for any abnormalities that may have prevented readmission.

Results

There were 195 readmissions (195 patients), of which 17 were due to mechanical complications. There was no evidence of fracture or abnormality appreciated on any of the reviewed PACU radiographs by either the reading radiologist or the senior authors. Assuming all fractures were noted on immediate, postoperative radiographs, the cost associated with identifying a single fracture in 2,415 patients was $1,072,260.

Conclusion

Routine radiographs in the recovery room after an uncomplicated primary TKA are not a reliable mechanism for preventing mechanical complications and do not alter patient care.

## Introduction

Total knee arthroplasty (TKA) is among the most commonly performed orthopedic procedures in the United States, totaling over 700,000 arthroplasty procedures in 2012 [1-2]. The annual rates of total joint arthroplasty continue to rise, with rates of TKA tripling between 1990 and 2002 [3-5]. Given our aging population, as well as the successful outcomes, arthroplasty procedures are expected to increase, with the demand for TKA expected to grow by 673% by 2030 [6-7].

Healthcare costs in the United States continue to increase and total joint arthroplasty is no exception, accounting for ten billion dollars annually, more than any other inpatient procedure [8-9]. In an effort to decrease cost, there has been an increased emphasis on alternative payment models as well as bundled payment plans. The initiation of these strategies has reduced hospital length of stay, readmission rate, rate of discharge to inpatient facilities, and the average cost for arthroplasty procedures, catalyzing a further discussion of strategies to decrease cost following arthroplasty procedures [10].

Obtaining radiographs in the post-anesthesia care unit (PACU) has been the standard of care at most hospitals. Previously, many surgeons also obtained additional radiographs prior to discharge, a practice that has been widely discontinued [11]. Additionally, immediate postoperative radiographs are often of poor quality secondary to bulky dressings, rotated images, and difficulty with patient compliance. Previous studies have scrutinized the utility of obtaining immediate postoperative radiographs in partial knee arthroplasty [12], TKA in a limited sample [13], as well as TKA within the first 60 days postoperatively [14]. Concerns over the utility of immediate postoperative radiographs in uncomplicated primary TKA were the basis of our investigation. The primary outcome of this study was to examine the utility of immediate, postoperative radiographs in detecting mechanical complications and their effect on postoperative management following uncomplicated TKA. Secondarily, we aimed to evaluate the cost-effectiveness of PACU radiographs by analyzing the cost of radiographs in our patient cohort. 

## Materials and methods

A retrospective review of all cemented and uncemented TKA performed at a suburban, regional medical center between January 2016 to June 2019 was performed. Patients included in the study consisted of those who underwent a primary cemented or uncemented TKA for arthritic conditions, refractory to conservative management. Patients were excluded if they had a revision TKA, unicondylar knee arthroplasty, or a conversion from unicondylar to TKA. International Classification of Diseases, Tenth Revision (ICD-10) codes were used to track any readmissions within 90 days, and a chart review was performed in those patients to determine if the readmission was for mechanical complications (fracture, dislocation, instability, or component loosening). Medical complications, such as wound issues, deep venous thrombosis, pulmonary embolism, and cardiopulmonary issues, were excluded. Postoperative radiographs of all patients readmitted for mechanical complications were reviewed by three of the authors (TAN, JNP, JD) to determine if any of the mechanical complications were visible on PACU radiographs and if there was any change in postoperative management that could have potentially avoided readmission. Additionally, the radiologist report was reviewed for the 17 readmitted patients, and any discrepancies between the reviews were recorded. Statistics were performed using Microsoft Excel (Microsoft Corporation, Redmond, WA).

## Results

The study group consisted of 4,864 consecutive, cemented TKA (4,319 patients) and 552 consecutive, uncemented TKA (511 patients) from January 2016 to June 2019. There was a total of 3,774 unilateral TKA and 545 bilateral TKA in the cemented group and a total of 470 and 41, respectively, in the uncemented group (Table 1).

**Table 1:** Volume and readmission rates for cemented and uncemented TKA TKA: total knee arthroplasty

	Unilateral TKA	Bilateral TKA	Total TKA	90-Day Readmissions	90-Day Readmission Rate
Cemented TKA	3774	545	4864	188	4.30%
Uncemented TKA	470	41	552	7	1.40%
Totals	4244	586	5416	195	3.60%

**Figure 1:**
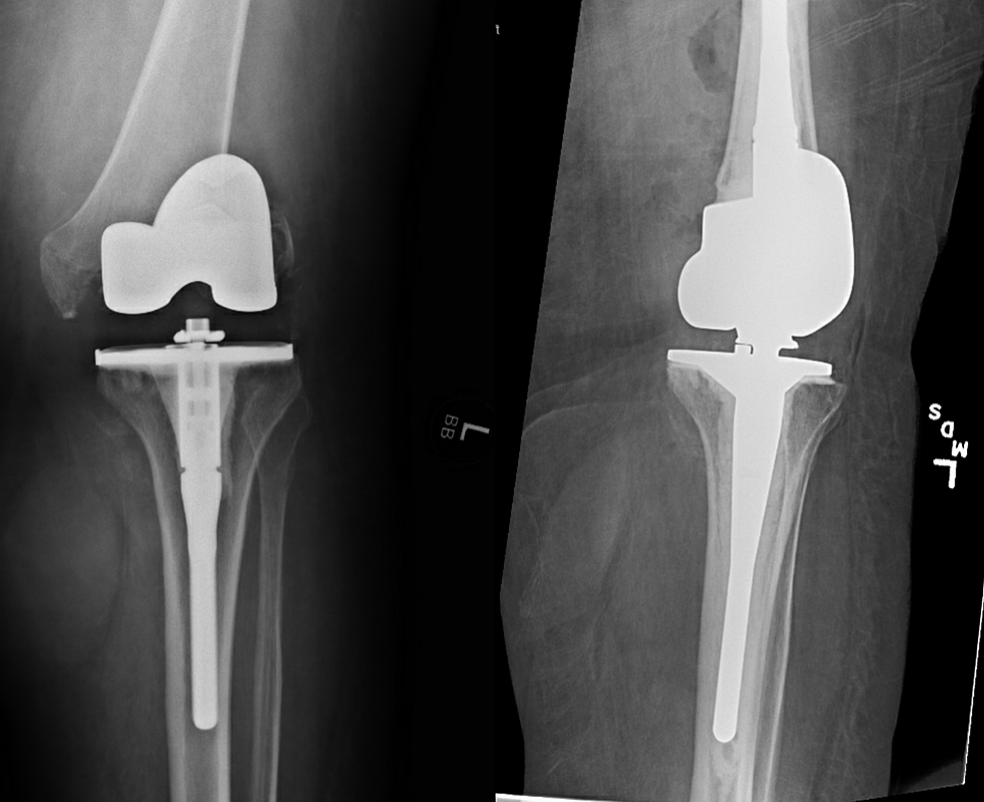
Readmission radiographs post primary total knee arthroplasty showing a periprosthetic distal femur fracture (left); status post revision total knee arthroplasty due to periprosthetic distal femur fracture (right)

**Figure 2:**
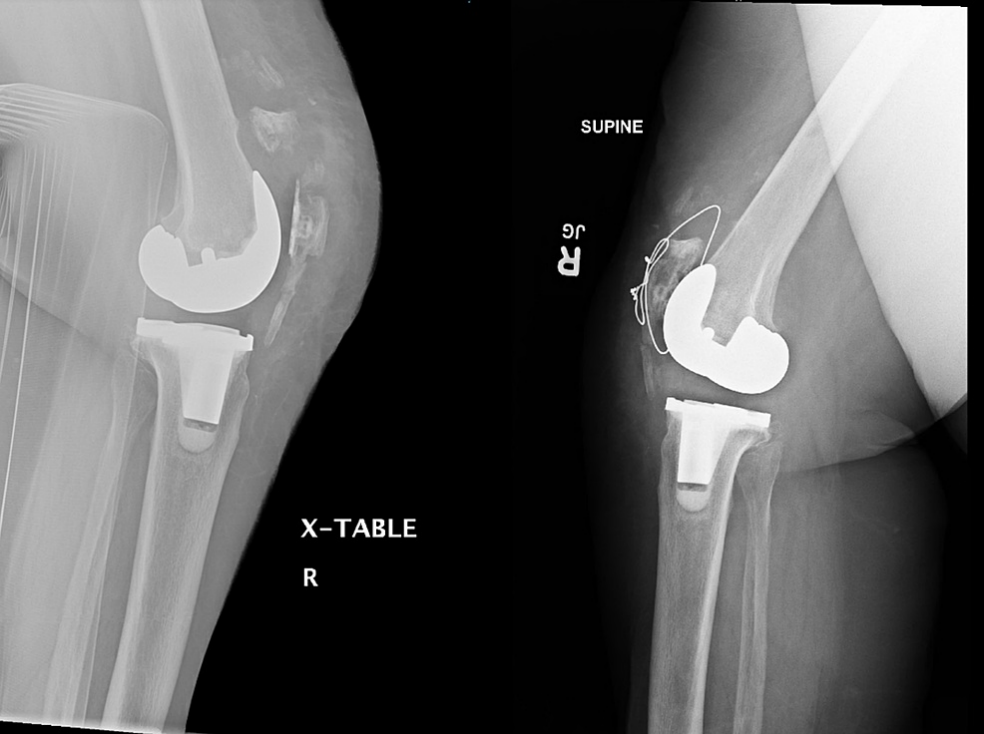
Readmission radiographs post primary total knee arthroplasty demonstrating a periprosthetic patella fracture (left); status post surgical fixation of periprosthetic patella fracture (right)

**Figure 3:**
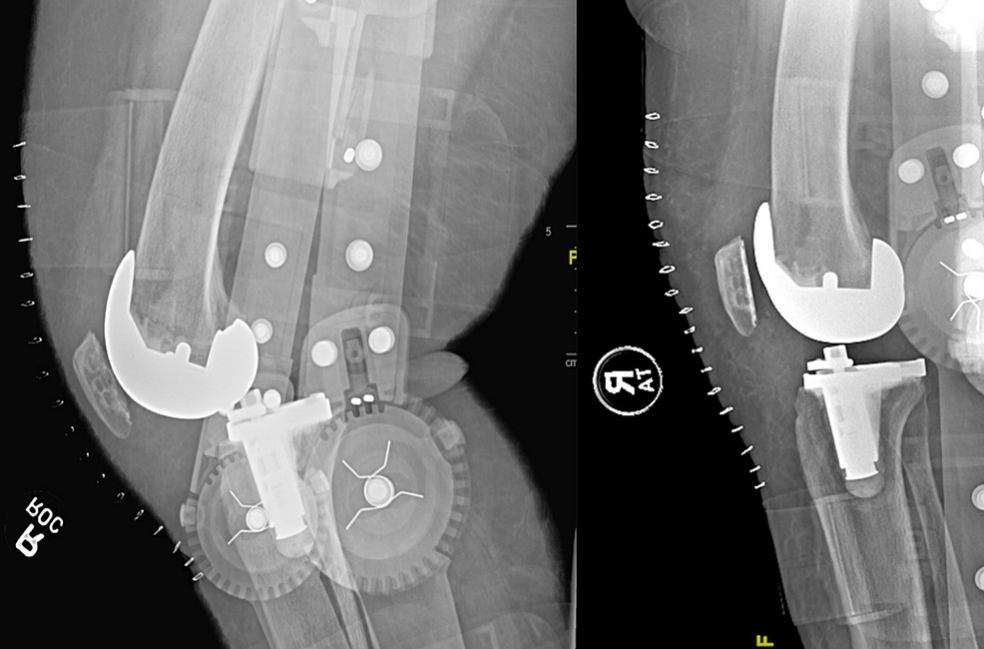
Readmission radiographs post primary total knee arthroplasty showing instability of the knee (left); revision total knee arthroplasty due to instability (right)

**Figure 4:**
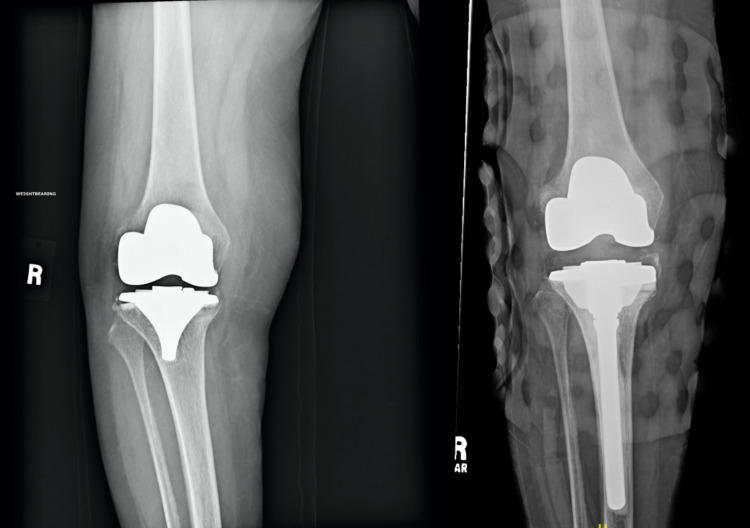
Readmission radiographs post primary total knee arthroplasty demonstrating instability of the tibial component (left); revision total knee arthroplasty for aseptic loosening (right)

There were 188 readmissions (188 patients) in the cemented TKA group, 17 of which were for mechanical complications. The remainder of the readmissions were for medical reasons. These complications included: 12 periprosthetic fractures (10 femur fractures (Figure 1)), two patella fractures (Figure 2), two cases of instability requiring revision (Figure 3), and three cases of mechanical loosening requiring revision (Figure 4). In the uncemented TKA group, there were seven readmissions (seven patients), none of which were for mechanical complications (Table 2).

**Table 2:** Mechanical complications for cemented and uncemented TKA TKA: total knee arthroplasty

	Readmissions	Mechanical Complications	% Mechanical Complications	Periprosthetic Fractures	Mechanical Instability	Mechanical Loosening
Cemented TKA	188 (4.35%)	17 (0.39%)	9.04%	12	2	3
Uncemented TKA	7 (1.37%)	0	0%	0	0	0
Totals	195	17	8.72%	12	2	3

There was no evidence of fracture or abnormality appreciated on any of the PACU radiographs reviewed by either the reading radiologist or the three aforementioned authors (TAN, JNP, JD). Eight of the 17 readmitted patients presented after a ground-level fall postoperatively and had no documented evidence of complication prior to the inciting event. In total, the incidence of periprosthetic fracture in the total cohort was 0.24%. None of the periprosthetic fractures were seen on initial postoperative imaging. The average time to revision was 36 days for periprosthetic fracture patients, 11 days for instability patients, and 67 days for patients with mechanical loosening.

At our institution, the patient charge for the performance and interpretation of PACU radiographs is $444 for unilateral TKA and $555 for bilateral TKA patients. The estimated radiographic expenditure for the entire study group, based on patient charges, is outlined in Table 3. The total patient charge for the PACU radiographs for all TKA patients in the cemented and uncemented groups was $2,209,566. This is significant, as none of the mechanical complications were identified on the PACU radiographs, questioning the justification for the patient charges.

**Table 3:** Patient charges associated with immediate postoperative imaging TKA: total knee arthroplasty

	Unilateral TKA Costs	Bilateral TKA Costs	Total Cost		
Cemented TKA	$1,675,656	$302,475	$1,978,131		
Uncemented TKA	$208,680	$22,755	$231,435		
Total	$1,884,336	$325,230	$2,209,566		

## Discussion

Value-based care models have forced hospitals and physicians to evaluate the cost-efficiency of each step in the healthcare process. The role of immediate postoperative radiographs for routine, primary TKA in the early recognition and prevention of postoperative complications is not well-defined in the literature. This is especially pertinent, as TKA procedures transition to the outpatient-only list for reimbursement with bundled care payment models. The present study calls into question the necessity of recovery room radiographs after uncomplicated primary TKA, as none of the mechanical complications were identified on immediate recovery room imaging. The elimination of routine postoperative imaging in TKA could have led to $2,209,566 in patient cost savings during our study period. Importantly, this value only accounts for the patient cost of the radiographs and their interpretation and does not account for other expenses such as extra time spent by the patient in the recovery room slowing down hospital throughput or the costs to hire additional, full-time radiology technicians to perform PACU radiographs.

The necessity of routine, postoperative imaging following uncomplicated knee arthroplasty has previously been evaluated in several smaller studies [12-14], but there is sparse literature evaluating the importance of postoperative radiographs and their effect on operative decision-making following uncomplicated TKA. In this study, we found routine radiographs did not alter or delay the clinical management of patients or prevent readmissions due to a mechanical complication. The average time to revision for all patients was 36 days for periprosthetic fracture, 11 days for instability, and 67 days for mechanical loosening.

Historically, obtaining immediate postoperative radiographs after TKA was considered the standard of care. Ververeli et al. evaluated 222 consecutive TKA and THA patients (124 cemented knees, 98 uncemented hips) to determine if immediate post-operative and pre-discharge radiographs would alter clinical management [11]. They reported no changes in postoperative management based on surgeon or radiologist review. Sambandam et al. performed a retrospective study on 220 patients who underwent TKA performed by one of two different surgeons [15]. Surgeon A required pre-discharge radiographs, while surgeon B performed weight-bearing radiographs six weeks postoperatively. The quality of the pre-discharge radiographs was deemed sufficient in only 58% of knees (65/112). A cost reduction of almost $220 per patient was found with the elimination of pre-discharge films. These studies support the concept that the exclusion of immediate postoperative radiographs following uncomplicated primary total joint arthroplasty does not alter the postoperative course and can lower overall costs in providing care for these patients.

Hassan et al. looked at 624 consecutive recovery room radiographs following TKA [16]. Only two patients had positive findings: a nondisplaced periprosthetic tibia fracture and an inferior pole patellar avulsion fracture. Neither of these patients needed additional treatment nor had a change in the postoperative mobilization protocol. Similarly, Glaser et al. analyzed the utility and quality of postoperative radiographs in 200 consecutive primary TKAs [17]. Of the 192 knees that had immediate postoperative radiographs, there was no change in postoperative management based on radiographs. Additionally, only 36% of radiographs were of adequate quality to serve as a baseline for future radiographs. They also analyzed 550 patients who had their first postoperative radiographs performed six weeks following surgery. They found no cases where radiographs taken earlier would have led to a deviation from routine postoperative protocols. Although it is out of the scope of this study, it does highlight the difficulty in obtaining quality postoperative radiographs due to multiple factors, including portable machines, surgical dressings, patient pain tolerance in the PACU, patients still under the effects of anesthesia, all of which further contribute to their decreased utility. In addition to immediate recovery room radiographs, Bessette et al. questioned the need for routine radiographs in asymptomatic patients in the initial two years after primary TKA [13]. In their study, 12 out of 263 TKA patients had abnormal radiographic findings two years after surgery. Three of the 12 patients underwent reoperation based on the radiographic findings. All three patients reported having atypical symptoms when their films were obtained. None of the remaining nine patients had symptoms that were attributable to any abnormal findings on radiographs; their management was not altered based on the radiographic finding.

Our current study also reflects a paradigm change seen in other orthopedic procedures. Longenecker et al. performed a retrospective review of 1366 consecutive, uncomplicated partial knee arthroplasties [12]. Patients were categorized into two groups based on patients that had PACU radiographs (n = 1184) and those that did not (n = 182). There were no reoperations based on radiographic findings in the PACU or at the first follow-up appointment. The authors concluded that forgoing PACU radiographs and obtaining them at the initial follow-up visit would result in cost reduction without detriment to patient care. The utility of postoperative radiographs has been challenged in other, common orthopedic procedures as well. Werner et al. reviewed the effectiveness of radiographs following routine anterior cruciate ligament (ACL) surgery [18]. Radiographs were read as normal by a musculoskeletal radiologist in 97.7% of patients, and there was no significant alteration to patient care based on imaging. In their study, a total of $336,683 ($562 per patient) was charged to patients for postoperative radiographs. They concluded that routine radiographs after uncomplicated ACL reconstruction may not be necessary and may represent a potential target for cost savings. Additionally, Stucken et al. saw similar results in their review of patients undergoing ACL reconstruction [19]. No complications were seen on postoperative radiographs as interpreted by the operative surgeon. The total cost of performing and interpreting radiographs was $14,490.80 for their patients.

There are several limitations to this study. First, due to the retrospective nature of the study, it has all of the inherent biases associated with retrospective studies. At our institution, there has been a shift away from obtaining routine postoperative radiographs by some surgeons. This led to a decrease in the total number of immediate postoperative radiographs available for inclusion in the analysis. However, given the large number of patients in this study who did have radiographs available, we believe our findings are representative of this patient population. Finally, it is possible that a patient may have had a mechanical complication and gone to a different hospital for care; however, given our role as a large tertiary referral center for complicated arthroplasty, we feel this number would be quite low, although not examined in our current study. Despite these limitations, our data suggest that there is a limited role for the routine use of immediate PACU radiographs following uncomplicated primary TKA and has significant cost-saving potential.

Due to these findings, the authors suggest obtaining postoperative radiographs following uncomplicated routine primary TKA during the first postoperative office visit. At this time, patients are more cooperative, can usually tolerate weight-bearing films, and can be better positioned for imaging for more adequate and accurate radiographs, which can be used as a baseline for future studies. Many surgeons obtain radiographs in the recovery room and again approximately two weeks later at the first postoperative visit with no change in postoperative management, calling into question the utility of these radiographs. Many surgeons obtain postoperative radiographs for immediate feedback so they can evaluate and critique their work. In the vast majority of cases, however, the patient’s postoperative care is not altered. With the lack of standardization in the quality and positioning of PACU radiographs, it may be more prudent to wait until the first postoperative office visit for these radiographs. Alternatively, PACU standardization protocols, as well as resident and radiology technician education on the adequacy of imaging at academic institutions, are other areas of improvement. However, if there is any concern during surgery or during the patient’s postoperative hospital course, radiographs can be obtained sooner. The present study builds on the growing body of literature, calling into question the utility and cost-effectiveness of immediate, postoperative recovery room radiographs in patients undergoing uncomplicated primary TKA.

## Conclusions

TKA is one of the most common procedures performed in the United States and, given the current healthcare environment, there has been a push for improving medical value in TKA. One aspect includes the need for routine radiographs in the postoperative recovery room. In this study, we found that routine radiographs after an uncomplicated primary TKA are not a reliable mechanism for identifying and preventing postoperative mechanical complications and did not alter postoperative management. The elimination of these routine radiographs leads to increased value and decreased costs in TKA. Further investigation is warranted, with randomized controlled trials, including additional cost-saving analyses.

## References

[1] Pabinger C, Lothaller H, Geissler A (2015). Utilization rates of knee-arthroplasty in OECD countries. Osteoarthritis Cartilage.

[2] Healthcare Cost and Utilization Project (HCUP (2014). Healthcare Cost and Utilization Project (HCUP). Nationwide Inpatient Sample (NIS). statistical brief # 186. https://www.hcup-us.ahrq.gov/reports/statbriefs/sb186-Operating-Room-Procedures-United-States-2012.pdf.

[3] Kurtz S, Mowat F, Ong K, Chan N, Lau E, Halpern M (2005). Prevalence of primary and revision total hip and knee arthroplasty in the United States from 1990 through 2002. J Bone Joint Surg Am.

[4] Maradit Kremers H, Larson DR, Crowson CS (2015). Prevalence of total hip and knee replacement in the united states. J Bone Joint Surg Am.

[5] Bini SA, Sidney S, Sorel M (2011). Slowing demand for total joint arthroplasty in a population of 3.2 million. J Arthroplasty.

[6] Kurtz S, Ong K, Lau E, Mowat F, Halpern M (2007). Projections of primary and revision hip and knee arthroplasty in the United States from 2005 to 2030. J Bone Joint Surg Am.

[7] Konopka JF, Lee YY, Su EP, McLawhorn AS (2018). Quality-adjusted life years after hip and knee arthroplasty. Health-related quality of life after 12,782 joint replacements. JB JS Open Access.

[8] Lavernia CJ, Drakeford MK, Tsao AK, Gittelsohn A, Krackow KA, Hungerford DS (1995). Revision and primary hip and knee arthroplasty. A cost analysis. Clin Orthop Relat Res.

[9] McLawhorn AS, Buller LT (2017). Bundled payments in total joint replacement: keeping our care affordable and high in quality. Curr Rev Musculoskelet Med.

[10] Dundon JM, Bosco J, Slover J, Yu S, Sayeed Y, Iorio R (2016). Improvement in total joint replacement quality metrics. Year one versus year three of the bundled payments for care improvement initiative. J Bone Joint Surg Am.

[11] Ververeli PA, Masonis JL, Booth RE, Hozack WJ, Rothman RH. (1996 (1996). Radiographic cost reduction strategy in total joint arthroplasty: a prospective analysis. J Arthroplasty.

[12] Longenecker AS, Kazarian GS, Boyer GP, Lonner JH (2017). Radiographic imaging in the postanesthesia care unit is unnecessary after partial knee arthroplasty. J Arthroplasty.

[13] Bessette MC, Amsdell SL, Giordano BD, Kates SL (2017). The utility of postoperative radiographs 2 years after primary total knee arthroplasty. J Arthroplasty.

[14] Murphy L, Helmick CG (2012). The impact of osteoarthritis in the United States. A population-health perspective. A population-based review of the fourth most common cause of hospitalization in U.S. adults. Orthop Nurs.

[15] Sambandam SN, Khanna V, Rohinikumar G, Mounasamy V (2017). Pre-discharge postoperative radiographs after primary total knee replacement: tradition or science?. Acta Orthop Belg.

[16] Hassan S, Wall A, Ayyaswamy B, Rogers S, Mills SP, Charalambous CP (2012). Is there a need for early post-operative x-rays in primary total knee replacements? Experience of a centre in the UK. Ann R Coll Surg Engl.

[17] Glaser D, Lotke P (2000). Cost-effectiveness of immediate postoperative radiographs after uncomplicated total knee arthroplasty: a retrospective and prospective study of 750 patients. J Arthroplasty.

[18] Werner BC, Burrus MT, Kew ME, Dempsey IJ, Gwathmey FW, Miller MD, Diduch DR (2016). Limited utility of routine early postoperative radiography after primary ACL reconstruction. Knee.

[19] Stucken C, Flato R, O'Hagan T (2015). Postoperative radiographs after ACL reconstruction are not cost-effective. Orthopedics.

